# Neutrophil extracellular trap markers predict thrombotic risk in patients with hyperhomocysteinemia: development and validation of a predictive model

**DOI:** 10.3389/fphys.2025.1695292

**Published:** 2025-12-10

**Authors:** Lifan Shao, Shujie Zhang, Zhenyu Wang, Chang’an Pei, Yanjie Jiang, Guangxin Cao

**Affiliations:** 1 School of Clinical Medical College, Shandong Second Medical University, Weifang, Shandong, China; 2 Weifang People’s Hospital, Shandong Second Medical University, Weifang, Shandong, China

**Keywords:** DVT, hyperhomocysteinemia, NEtosis, NETs, nomogram

## Abstract

**Background:**

Venous thromboembolic diseases pose a serious threat to human health, and their pathogenesis is closely associated with the abnormal activation of neutrophils and neutrophil extracellular traps (NETs). However, the mechanisms underlying the role of NETs in thrombosis related to Hyperhomocysteinemia (hHcy) remain unclear. In this study, we systematically investigated the correlation between NETs and hHcy-associated thrombosis by measuring key NETs markers (myeloperoxidase, MPO; citrullinated histone H3, CitH3) and cell-free DNA (cfDNA) levels. Based on multivariate analysis, we developed and validated a thrombotic risk prediction model integrating clinical indicators and NETs markers, providing novel biomarkers and an assessment tool for early clinical identification of high-risk patients.

**Methods:**

This study consecutively enrolled 394 hHcy patients who visited the Department of Vascular Surgery and Health Examination Center at Weifang People’s Hospital between November 2023 and April 2025. The cohort comprised 71 patients in the deep vein thrombosis (DVT) group and 323 in the non-DVT control group. We systematically collected baseline clinical characteristics and laboratory parameters from all participants, with DVT occurrence serving as the primary endpoint. Independent risk factors for DVT were identified through univariate and multivariate logistic regression analyses, which were subsequently used to construct a risk prediction model. To validate model reliability, internal validation was performed using the following approaches: The discriminative ability of the model was evaluated by analyzing the area under the curve (AUC) of the receiver operating characteristic (ROC) curve, calibration curves to assess consistency, and ecision curve analysis to determine clinical utility. These comprehensive validation methods were applied to both training and validation cohnrts to rigorously test the model’s predictive performance.

**Result:**

Laboratory test results revealed significantly elevated levels of NETs-related markers in the peripheral blood of DVT patients, including Myeloperoxidase-Deoxyribonucleic Acid (MPO-DNA), CitH3, cfDNA, and absolute neutrophil count. Multivariate logistic regression analysis confirmed that MPO-DNA (Odds Ratio, OR = 11.58, 95% CI: 3.75–35.73, Probability Value, p < 0.001), CitH3 (OR = 1.11, 95% CI: 1.05–1.17, p < 0.001), cell-free DNA (OR = 1.02, 95% Confidence Interval, 95% CI: 1.01–1.02, p < 0.001), and neutrophil (OR = 1.67, 95% CI: 1.14–2.44, p = 0.008) were all independent risk factors for DVT in hHcy patients. The risk prediction model constructed based on these factors demonstrated excellent discriminative performance, with area under the receiver operating characteristic curve values of 0.93 (95% CI: 0.88–0.99) in the training cohort and 0.96 (95% CI: 0.88–1.00) in the validation cohort.

**Conclusion:**

This study demonstrates that hHcy-associated thrombosis is significantly correlated with elevated levels of NETs. Based on these findings, we have developed a novel non-invasive thrombotic risk prediction model that can effectively identify high-risk hHcy patients.

## 1 Introduction

Deep vein thrombosis (DVT) refers to a venous circulatory disorder characterized by abnormal blood coagulation within deep veins, leading to vascular occlusion. This condition predominantly affects the lower extremities and represents one of the most prevalent peripheral vascular diseases, with a global incidence rate of approximately 1.6‰ (Albricker et al., 2022). The high incidence and severe clinical consequences of DVT underscore the critical importance of early intervention, as timely treatment can significantly reduce both complication rates and mortality risk (Caldeira et al., 2015).

Neutrophil extracellular traps (NETs) play a central role in pathological thrombus formation through a multi-step cascade mechanism: First, NETs initiate a prothrombotic microenvironment by mediating the recruitment and activation of platelets and neutrophils on the endothelial surface. Second, neutrophils release NETs structures containing histones and proteases via the NETosis process, and these components directly activate the coagulation cascade, promoting fibrin network formation (Gould et al., 2015; Kimball et al., 2016; Carestia et al., 2016). Studies have demonstrated that NETs not only induce platelet aggregation and provide a scaffold structure, but also influence thrombus stability through their interaction with fibrin (Fuchs et al., 2010). As pivotal effector cells of innate immunity, neutrophils contribute to immune defense through multiple mechanisms, including phagocytosis, antimicrobial protein release, reactive oxygen species (ROS) generation, and NETs formation (Yao et al., 2023; Huang et al., 2011). Notably, a bidirectional interaction exists between activated neutrophils and endothelial cells: neutrophils can induce endothelial cell damage (Wang and Liu, 2021; Rabinovitch, 2016), while activated endothelial cells in turn promote NETs release (Dourado et al., 2022), forming a positive feedback loop that exacerbates thrombus formation.

Recent studies have established that platelet activation serves as a critical regulator in NETs formation and thrombotic processes (Kim and Jenne, 2016). Upon vascular endothelial injury, exposed collagen initiates platelet adhesion and activation, prompting the release of mediators including adenosine diphosphate (ADP) and Thromboxane A2 (TXA2) which facilitate platelet aggregation and primary thrombus formation (Longstaff and Kolev, 2015). Activated platelets not only induce NETs release from neutrophils, but these NETs reciprocally provide a structural scaffold for platelet aggregation while interacting with fibrin to modulate thrombus stability (Fuchs et al., 2010), thereby creating a self-amplifying “platelet-NETs-endothelial cell” axis. This pathogenic cascade is further augmented by endothelial-derived factors such as IL-8 and ROS that promote additional NETs generation (Elaskalani et al., 2018; Wagner, 2005). Histopathological evidence from DVT specimens demonstrates distinct co-localization of CitH3 with von Willebrand factor (vWF) and platelet aggregates, mechanistically linking NETs-mediated histone citrullination to thrombus development (Savchenko et al., 2014). Emerging evidence reveals that NETs drive thrombus progression through coordinated pathogenic mechanisms: by activating the MPO/H_2_O_2_-TLR4/NF-κB signaling pathway to induce pro-inflammatory endothelial responses while simultaneously disrupting endothelial barrier function via excessive ROS production (Aldabbou et al., 2016), thereby creating a self-reinforcing pathological cycle that exacerbates venous thromboembolic events.

At the molecular level, both elevated plasma homocysteine and active NETs formation constitute independent risk factors for thromboembolic disorders. Homocysteine, a non-proteinogenic sulfhydryl-containing amino acid derived from methionine metabolism and a biological homolog of cysteine, plays a pivotal role in vascular pathology. Hyperhomocysteinemia (hHcy), defined as a pathological elevation of plasma total homocysteine concentration (Sen et al., 2010), induces a prothrombotic state through multiple mechanisms: it triggers elevated inflammatory cytokine production, alters DNA methylation patterns (Hermann and Sitdikova, 2021), and critically impairs endothelial cell function. Substantial clinical evidence has established hHcy as a significant risk factor for both atherosclerosis and thromboembolic diseases (Austin et al., 2004; den Heijer et al., 2007), with particular relevance to DVT and pulmonary embolism risk stratification. While the individual contributions of NETs and hHcy to thromboembolism are well-documented, current research reveals a critical knowledge gap regarding their potential synergistic interactions and combined prognostic significance in thrombotic disorders.

This study aims to investigate the correlation between NETs and hyperhomocysteinemia-associated venous thromboembolism, with the ultimate goal of developing novel clinical therapeutic strategies for patients with DVT.

## 2 Materials and methods

### 2.1 Study population

This retrospective study enrolled 394 subjects with hyperhomocysteinemia from the Department of Vascular Surgery at Weifang People’s Hospital between November 2023 and April 2025, including 71 hHcy-associated DVT patients and 323 non-hHcy DVT controls. The study protocol was approved by the Institutional Review Board of Weifang People’s Hospital (Ethics Approval No.: KYLL20241008-20), with waiver of informed consent granted due to the retrospective nature of the investigation. The study was conducted in accordance with the Declaration of Helsinki (as revised in 2013).

The inclusion criteria comprised: (1) age >18 years; (2) radiologically confirmed lower extremity DVT via diagnostic modalities including color Doppler ultrasound, CT venography, or conventional venography; (3) elevated D-dimer levels >1.00 μg/mL; (4) no prior anticoagulant therapy before hospitalization; and (5) serum homocysteine (Hcy) levels >17 μmol/L for the hHcy group. Exclusion criteria included: (1) incomplete medical records or loss to follow-up; (2) comorbid conditions such as active malignancies, infections, or autoimmune diseases; (3) pregnancy or lactation status; and (4) chronic use of medications for other systemic disorders.

### 2.2 Study endpoints

The primary endpoint of this study was lower extremity DVT, with positive findings on Doppler ultrasonography serving as the diagnostic gold standard (D'Oria et al., 2025; Grant et al., 2024).

### 2.3 Laboratory methods and statistical analysis

Sample Preparation: Venous blood was collected at admission using standard phlebotomy procedures. For serum separation, non-anticoagulated blood samples were centrifuged and the supernatant serum was aliquoted and stored at −80 °C until subsequent analyses.

Serum Myeloperoxidase-Deoxyribonucleic Acid (MPO-DNA) and citrullinated histone H3, (CitH3) Assays: MPO-DNA complexes in serum were quantified using a human NETs (MPO-DNA) ELISA kit (Xiamen LunChangShuo Biotechnology, China). After 120-min temperature equilibration at 25 °C–28 °C, 50 μL of serum samples were loaded into 96-well plates followed by addition of 100 μL horseradish peroxidase (HRP)-conjugated detection antibody. The plates were sealed and incubated at 37 °C for 60 min. Color development was achieved using TMB substrate (30-min dark incubation), with optical density measured at 450 nm. CitH3 levels were determined using a commercial ELISA kit (ELK Biotechnology, Wuhan, China) following manufacturer’s protocol. Briefly, 100 μL serum samples were incubated with 100 μL biotinylated antibody and 100 μL HRP-conjugated detection antibody for 50 min at 37 °C. After 20-min TMB substrate reaction in darkness, absorbance was read at 450 nm.

Cell-Free DNA (cfDNA) Quantification: Serum cfDNA concentrations were measured using the PicoGreen® dsDNA Quantification Kit (Solarbio, Beijing). A standard curve was generated using serial dilutions of reference DNA. Each well received 100 μL serum sample or standard mixed with 100 μL PicoGreen working solution. After vortex mixing (2000 rpm, 30 s) and 5-min dark incubation at room temperature, fluorescence intensity was measured (excitation 480 nm/emission 520 nm). All assays were performed blinded to clinical data within reagent expiration periods.

Statistical Analysis: Continuous variables were compared using Student's t-test or Mann-Whitney U test as appropriate. Categorical variables were analyzed by χ^2^ or Fisher’s exact tests. Univariate and multivariate logistic regression identified independent predictors, with two-tailed p < 0.05 considered statistically significant. All analyses were performed using R software (version 4.5.1).

### 2.4 Development and validation of the clinical prediction model

Based on previous research on DVT risk factors (Li Y. et al., 2022), we retrospectively analyzed clinical data including demographic characteristics (age, sex, BMI), hematological parameters (neutrophil count, lymphocyte count, platelet count, neutrophil-to-lymphocyte ratio), and NETs-specific biomarkers (MPO-DNA, CitH3, cfDNA). This study included 394 patients with hyperhomocysteinemia to develop a DVT prediction model, randomly divided into training and validation cohnrts in a 7:3 ratio (276 and 118 patients, respectively).

The analysis involved three key steps: First, univariate logistic regression was performed in the training cohort to assess the predictive capacity of clinical factors for lower extremity DVT. Subsequently, variables demonstrating statistically significant differences (p < 0.05) in the univariate analysis were incorporated into the multivariate analysis. Those variables that maintained statistical significance (p < 0.05) in the multivariate analysis were ultimately defined as independent predictive factors in this study. Finally, independent predictors identified through multivariate logistic regression were used to construct a predictive nomogram.

The model was systematically evaluated in both the training and validation cohorts using three complementary approaches: receiver operating characteristic (ROC) curve analysis to determine discriminative ability, quantified by the area under the curve (AUC). Calibration curves to assess prediction accuracy, and decision curve analysis (DCA) to examine clinical utility. This comprehensive validation strategy ensured robust assessment of the nomogram’s predictive value for DVT risk stratification in hyperhomocysteinemia patients.

## 3 Result

### 3.1 Baseline characteristics analysis

#### 3.1.1 Characteristics of study population

The baseline characteristics of all 394 enrolled subjects (71 DVT patients and 323 non-DVT controls) are summarized in Table 1. The DVT group (mean age 60.03 ± 14.06 years) and non-DVT group (mean age 59.26 ± 12.80 years) showed comparable age distributions (p > 0.05), with similar gender proportions (56.34% male in DVT group vs. 59.44% in controls, p > 0.05). These results confirm balanced demographic characteristics between the two groups at baseline.

**TABLE 1:** Demographic data of patients.

Variables	Total (n = 394)	Non-DVT (n = 323)	DVT (n = 71)	*p*
Age^a^	59.41 ± 13.02	59.27 ± 12.80	60.03 ± 14.06	0.657
Gender				0.630
Male	232 (58.88)	192 (59.44)	40 (56.34)	
Female	162 (41.12)	131 (40.56)	31 (43.66)	
Hypertension				0.583
Yes	277 (70.30)	229 (70.90)	48 (67.61)	
NO	117 (29.70)	94 (29.10)	23 (32.39)	
Diabetes				**<0.001**
Yes	310 (78.68)	265 (82.04)	45 (63.38)	
No	84 (21.32)	58 (17.96)	26 (36.62)	
BMI (kg/m^2^)^a^	24.37 ± 3.52	24.09 ± 3.40	25.63 ± 3.80	**<0.001**
Lymphocyte (10^9^/L)^a^	1.88 ± 0.75	1.89 ± 0.76	1.84 ± 0.73	0.562
Neutrophil (10^9^/L)^a^	4.15 ± 1.46	3.97 ± 1.26	4.96 ± 1.95	**<0.001**
Platelet (10^9^/L)^a^	210.46 ± 81.40	209.73 ± 80.54	213.80 ± 85.74	0.703
NLR^a^	2.56 ± 1.43	2.42 ± 1.24	3.21 ± 1.99	**0.002**
D-dimer (μg/mL)^a^	1.72 ± 3.30	0.64 ± 0.70	6.63 ± 5.38	**<0.001**
MPO-DNA (O.D.)^a^	0.79 ± 0.59	0.64 ± 0.37	1.44 ± 0.89	**<0.001**
CitH3 (ng/mL)^a^	23.11 ± 11.27	21.05 ± 7.75	32.51 ± 18.10	**<0.001**
CfDNA (ng/mL)^a^	330.50 ± 109.98	303.30 ± 67.16	454.23 ± 167.97	**<0.001**

^a^ All data of this type are presented as mean ± standard deviation.

p < 0.05 was considered statistically significant.

Values in bold represent statistically significant results (p < 0.05).

BMI, body mass index; NLR, neutrophil–lymphocyte ratio; MPO-DNA, Myeloperoxidase-DNA complex; CitH3, Citrullinated Histone H3; cfDNA, Cell-free DNA.

**FIGURE 1:**
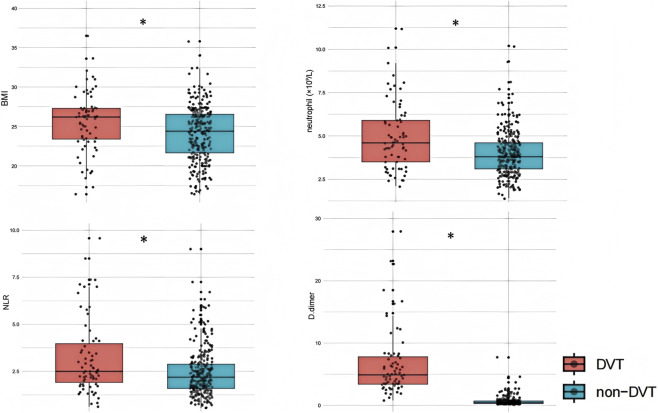
Statistical analysis of laboratory data after admission to hospital between the two study groups. *p < 0.05; NLR, neutrophil–lymphocyte ratio.

Comparative analysis revealed significant differences between DVT and non-DVT groups across multiple parameters (Table 1). The DVT group demonstrated substantially higher BMI levels and greater prevalence of diabetes mellitus (both p < 0.001). Furthermore, marked elevations were observed in neutrophil count (p < 0.001), neutrophil-to-lymphocyte ratio (NLR, p = 0.002), and D-dimer levels (p < 0.001; Figure 1). Notably, all three NETosis biomarkers showed highly significant increases in the DVT group: MPO-DNA complexes (p < 0.001), CitH3 (p < 0.001), and cfDNA (p < 0.001). All reported comparisons achieved statistical significance at p < 0.05 threshold.

### 3.2 Predictive modeling

#### 3.2.1 Baseline data analysis

The study population of 394 participants was randomly allocated into training (n = 276, 70%) and validation (n = 118, 30%) cohnrts, as shown in Table 2. The training cohnrt had a mean age of 59.41 ± 13.02 years with 159 males (57.61%), including 50 DVT cases (18.16%), while the validation cohnrt showed comparable demographics with a mean age of 60.00 ± 13.79 years, 73 males (61.86%), and 21 DVT cases (17.80%). Both groups demonstrated balanced baseline characteristics with no significant differences in age, sex distribution, or DVT prevalence (all p > 0.05), confirming appropriate cohort partitioning for model development and validation.

**TABLE 2:** Statistical characteristics of the training and validation cohnrts.

Variables	Total (n = 394)	Training cohnrt (n = 276)	Validation cohnrt (n = 118)	*p*
Age^a^	59.41 ± 13.02	59.15 ± 12.70	60.00 ± 13.79	0.555
Gender				0.432
Male	232 (58.88)	159 (57.61)	73 (61.86)	
Female	162 (41.12)	117 (42.39)	45 (38.14)	
BMI (kg/m^2^)				0.167
<24	169 (42.89)	112 (40.58)	57 (48.31)	
24–28	179 (45.43)	127 (46.01)	52 (44.07)	
≥28	46 (11.68)	37 (13.41)	9 (7.63)	
Hypertension				0.637
Yes	277 (70.30)	196 (71.01)	81 (68.64)	
NO	117 (29.70)	80 (28.99)	37 (31.36)	
Diabetes				0.055
Yes	310 (78.68)	210 (76.09)	100 (84.75)	
No	84 (21.32)	66 (23.91)	18 (15.25)	
Lymphocyte (10^9^/L)^a^	1.88 ± 0.75	1.87 ± 0.70	1.91 ± 0.87	0.693
Neutrophil (10^9^/L)^a^	4.15 ± 1.46	4.19 ± 1.48	4.06 ± 1.39	0.419
Platelet (10^9^/L)^a^	210.46 ± 81.40	211.18 ± 78.07	208.79 ± 89.04	0.790
NLR^a^	2.56 ± 1.43	2.57 ± 1.45	2.54 ± 1.40	0.876
D-dimer (μg/mL)^a^	1.72 ± 3.30	1.73 ± 3.31	1.69 ± 3.28	0.920
MPO-DNA (O.D.)^a^	0.79 ± 0.59	0.79 ± 0.60	0.79 ± 0.57	0.974
CitH3 (ng/mL)^a^	23.11 ± 11.27	23.47 ± 12.28	22.28 ± 8.42	0.339
CfDNA (ng/mL)^a^	330.50 ± 109.98	332.64 ± 107.12	325.49 ± 116.70	0.555

^a^ All data of this type are expressed as mean ± standard deviation (mean ± SD).

BMI, body mass index; NLR, neutrophil–lymphocyte ratio. MPO-DNA, Myeloperoxidase-DNA complex; CitH3, Citrullinated Histone H3; cfDNA: Cell-free DNA.

#### 3.2.2 Identification of independent predictors

Univariate logistic regression analysis identified several significant associations with DVT risk: BMI≥28 kg/m^2^ (Odds ratio, OR = 2.87, 95% Confidence interval, 95%CI = 1.01–8.18, p = 0.049), diabetes mellitus (OR = 3.13, 95%CI = 1.44–6.84, p = 0.004), elevated MPO-DNA (OR = 9.40, 95%CI = 4.21–20.98, p < 0.001), CitH3 (OR = 1.08, 95%CI = 1.04–1.12, p < 0.001), cell-free DNA (OR = 1.01, 95%CI = 1.01–1.02, p < 0.001), neutrophil count (OR = 1.47, 95%CI = 1.16–1.87, p = 0.002), and NLR (OR = 1.38, 95%CI = 1.11–1.72, p = 0.004) (Table 3). Subsequent multivariate analysis incorporating all univariately significant predictors (p < 0.05) demonstrated that MPO-DNA (OR = 11.58, 95%CI = 3.75–35.73, p < 0.001), CitH3 (OR = 1.11, 95%CI = 1.05–1.17, p < 0.001), cell-free DNA (OR = 1.02, 95%CI = 1.01–1.02, p < 0.001), and neutrophil count (OR = 1.67, 95%CI = 1.14–2.44, p = 0.008) emerged as independent predictors of DVT (Table 3).

**TABLE 3:** Univariate and multivariate logistic regression analyses of thrombosis-related factors in patients with hHcy.

Variables	Univariable		Multivariable	
	*P*	OR (95%CI)	*p*	OR (95%CI)
Age	0.418	1.01 (0.98–1.04)		
Gender				
Male		1.00 (Reference)		
Female	0.236	1.57 (0.74–3.31)		
BMI (kg/m^2^)				
<24		1.00 (Reference)		
24–28	0.452	1.38 (0.59–3.22)		
≥28	**0.049**	2.87 (1.01–8.18)	0.637	1.82 (0.15–21.59)
Hypertension				
Yes		1.00 (Reference)		
NO	0.762	0.88 (0.39–1.98)		
Diabetes				
Yes		1.00 (Reference)		
No	**0.004**	3.13 (1.44–6.84)	0.132	2.89 (0.73–11.51)
Lymphocyte (10^9^/L)	0.892	0.96 (0.57–1.63)		
Neutrophil (10^9^/L)	**0.002**	1.47 (1.16–1.87)	**0.008**	1.67 (1.14–2.44)
Platelet (10^9^/L)	0.567	1.00 (1.00–1.01)		
NLR	**0.004**	1.38 (1.11–1.72)	0.099	2.16 (0.87–5.37)
MPO-DNA (O.D.)	**<0.001**	9.40 (4.21–20.98)	**<0.001**	11.58 (3.75–35.73)
CitH3 (ng/mL)	**<0.001**	1.08 (1.04–1.12)	**<0.001**	1.11 (1.05–1.17)
CfDNA (ng/mL)	**<0.001**	1.01 (1.01–1.02)	**<0.001**	1.02 (1.01–1.02)

Values in bold represent statistically significant results (p < 0.05).

BMI, body mass index; NLR, neutrophil–lymphocyte ratio. MPO-DNA, Myeloperoxidase-DNA complex; CitH3, Citrullinated Histone H3; cfDNA, Cell-free DNA.

#### 3.2.3 Model development and validation

**FIGURE 2:**
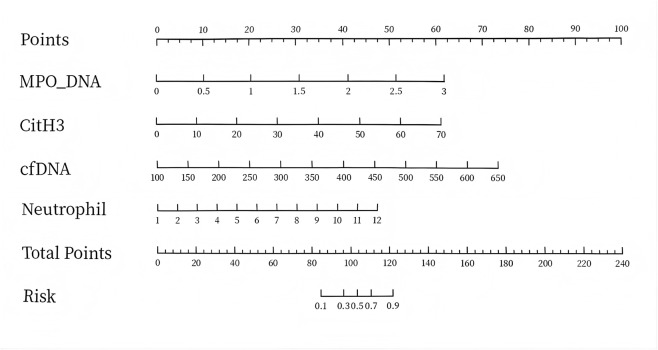
Nomogram Abbreviation: MPO-DNA, Myeloperoxidase-DNA complex; CitH3, Citrullinated Histone H3; cfDNA, Cell-free DNA.

**FIGURE 3:**
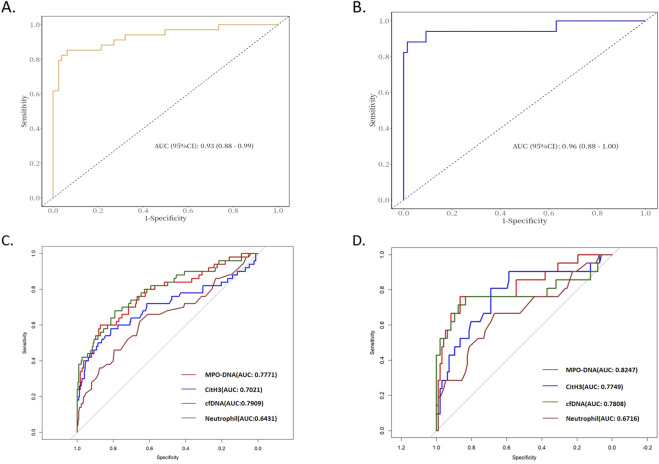
ROC curves of the model and individual predictive factors for thrombosis prediction in the training and validation cohnrts. **(A)** ROC of the model for DVT prediction in the training cohnrt; **(B)** ROC of the model for DVT prediction in the validation cohnrt; **(C)** ROC of individual model factors for DVT prediction in the training cohnrt; **(D)** ROC of individual model factors for DVT prediction in the validation cohnrt. Abbreviation: MPO-DNA, Myeloperoxidase-DNA complex; CitH3, Citrullinated Histone H3; cfDNA, Cell-free DNA.

**FIGURE 4:**
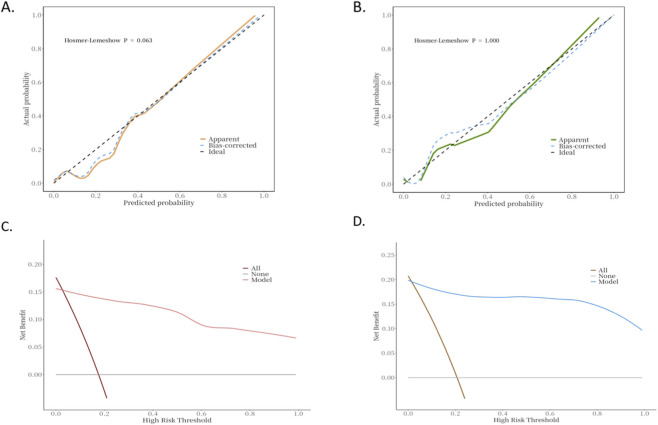
Calibration and decision curve analysis of the model in the training and validation cohorts. **(A)** Calibration curve of the predictive model for DVT risk assessment in hHcy patients (training cohnrt). **(B)** Calibration curve of the predictive model for DVT risk assessment in hHcy patients (validation cohnrt). **(C)** Decision curve analysis of the predictive model for DVT risk assessment in hHcy patients (training cohnrt). **(D)** Decision curve analysis of the predictive model for DVT risk assessment in hHcy patients (validation cohnrt).

A nomogram was constructed based on variables that remained statistically significant in the multivariate analysis (Figure 2). The model’s predictive performance was subsequently validated in both the training and validation cohorts. The results demonstrated excellent discriminative ability, with AUC of 0.93 (95% CI: 0.88–0.99) in the training cohnrt and 0.96 (95% CI: 0.88–1.00) in the validation cohnrt, outperforming individual predictive factors (Figure 3). Furthermore, calibration curves indicated strong agreement between predicted and observed probabilities, while DCA confirmed the model’s clinical utility across a wide range of risk thresholds (Figure 4). These findings collectively support the robust predictive performance of the nomogram for DVT risk stratification.

## 4 Discussion

This study is the first to investigate the association between NETs-associated regulatory proteins (MPO, CitH3) and cell-free DNA with thrombosis in hHcy, while developing and validating a clinically applicable prediction model for identifying high-risk patients. Key findings revealed significantly elevated levels of MPO-DNA complexes, CitH3, cell-free DNA, and neutrophil counts in the peripheral blood of hHcy-associated DVT patients compared to controls. Both univariate and multivariate analyses consistently identified MPO-DNA (OR = 11.58), CitH3 (OR = 1.11), cell-free DNA (OR = 1.02), and neutrophil count (OR = 1.67) as independent predictors of DVT. These four biomarkers were subsequently incorporated into a novel predictive nomogram that demonstrated excellent discriminative capacity (AUC 0.93–0.96) and clinical utility through comprehensive validation.

Studies demonstrated that hHcy not only activates platelets but also enhances NETs formation (Zhao et al., 2021). Mechanistically, hHcy impairs vascular function and exacerbates chronic inflammation through the release of damage-associated molecular patterns such as high-mobility group box-1 (HMGB-1), thereby worsening cardiovascular outcomes (Gadanec et al., 2023). Furthermore, HMGB-1 mediates intercellular communication by facilitating platelet-neutrophil interactions through surface receptor signaling (Vogel et al., 2015). Specifically, activated platelets transmit signals to neutrophil receptors, triggering neutrophil activation, aggregation, and subsequent NET release at sites of vascular injury (Maugeri et al., 2014).

Our study identified MPO-DNA (OR = 11.58, p < 0.001) as an independent predictor of DVT, consistent with the findings of Rena et al. (Rena et al., 2024). MPO, a product of neutrophil-mediated inflammatory responses (Khanna et al., 2018), has been extensively documented to participate in the pathogenesis of various diseases including coronary atherosclerosis (Trentini et al., 2020), stroke (Kim et al., 2019), neurodegenerative disorders (Wu et al., 2020), and malignancies (Rymaszewski et al., 2014). Subsequent research by Thålin et al. revealed that NETs contribute to DVT formation, with MPO serving as a crucial structural component of NETs (Thålin et al., 2018). Mechanistically, activated MPO mediates oxidative DNA modification, leading to the formation of procoagulant MPO-DNA complexes. These complexes interact with platelet surface receptors to promote platelet activation and subsequent release of prothrombotic factors such as platelet factor 4 (PF4) and thromboxane A2, thereby amplifying the coagulation cascade. Furthermore, MPO-DNA modulates erythrocyte function by enhancing aggregation and deformability, ultimately increasing blood viscosity to favor thrombus formation (Han et al., 2022). The prothrombotic effects are further augmented through MPO-DNA-mediated inflammatory responses (Li B. et al., 2022). Notably, Yan et al. demonstrated significantly reduced MPO-DNA levels in statin-treated patients (Yan et al., 2025), highlighting the therapeutic potential of targeting NETs in DVT management. However, the clinical efficacy and safety of such interventions require further validation through randomized controlled trials.

Consistent with the findings of Li et al. (Li Y. et al., 2022), our study demonstrated that CitH3 (OR = 1.11, p < 0.001) serves as an independent predictor of DVT. CitH3, a post-translationally modified form of histone H3, is normally localized within neutrophil nuclei but can be released into circulation during NETosis (Thålin et al., 2018; Wang et al., 2023). Current evidence identifies CitH3 as a core structural component of NETs (Neeli et al., 2008). Thålin et al. established CitH3’s diagnostic and prognostic value as a plasma biomarker for thrombosis, with peripheral blood CitH3 levels showing critical significance for DVT diagnosis and outcome prediction (Thålin et al., 2018). This correlation was further quantified by Mauracher et al., whose research revealed a 13% increased risk of venous thromboembolism per 100 ng/mL elevation in CitH3 levels (Mauracher et al., 2018).

The present study identified significantly elevated levels of cfDNA (OR = 1.02, p < 0.001) in the peripheral blood of DVT patients compared to controls, establishing it as an independent predictor of deep vein thrombosis - a finding consistent with previous reports by Liu et al. (Liu L. et al., 2021). As extracellular fragments of double-stranded DNA, cfDNA serves as both the structural backbone of NETs (Brinkmann et al., 2004) and a characteristic NETs biomarker (Sur Chowdhury et al., 2016), originating primarily from cell death and NETosis. Mechanistic studies demonstrate that cfDNA promotes thrombus formation through multiple pathways: (1) direct platelet activation leading to granular content release (Fuchs et al., 2010), (2) TLR9-mediated activation of monocytes/macrophages via Toll-like receptor signaling, which exacerbates inflammatory responses that potentiate thrombosis (Hoeter et al., 2023). These findings collectively elucidate the pivotal role of cfDNA in venous thromboembolism pathogenesis.

Our study identified neutrophil count as a significant independent predictor of DVT (OR = 1.67, p = 0.008), consistent with previous reports by Liu et al. (2020) which demonstrated that while inflammatory markers like neutrophil-to-lymphocyte ratio (NLR) showed no independent association with postoperative DVT, absolute neutrophil count emerged as an independent risk factor. Neutrophils play a pivotal role in venous thromboembolism as the most abundant leukocyte population in venous thrombi (Yao et al., 2023). These cells promote thrombus formation through multiple mechanisms, including activation of the NF-κB signaling pathway that induces endothelial cells to produce procoagulant factors (Guan et al., 2025). When neutrophil activation reaches a critical threshold, they interact with coagulation factor XII (FXII) and release NETs to directly facilitate DVT development (Yao et al., 2023). Furthermore, the work of Poddar et al. (Poddar et al., 2001) revealed that Hcy exacerbates this process by modulating interleukin-8 (IL-8) expression, triggering inflammatory responses that enhance neutrophil recruitment and activation, thereby creating a prothrombotic microenvironment. These findings collectively underscore neutrophils’ central role in venous thrombosis through both cellular and molecular pathways.

We developed a predictive model incorporating these independent risk factors, which demonstrated excellent predictive performance in both the training (AUC = 0.93) and validation (AUC = 0.96) cohorts, surpassing the predictive accuracy of previous DVT risk models (e.g., AUC = 0.757 in Yin Li’s study and AUC = 0.779 in Lei Liu’s study). Several factors may account for this superior performance: First, our relatively large sample size (n = 394) provided greater statistical power and reproducibility. Second, unlike previous single-factor approaches, our model comprehensively evaluated multiple NETs-related biomarkers (MPO-DNA, CitH3, and cfDNA) in the peripheral blood of hHcy patients. However, the clinical applicability of this prediction model requires further validation through large-scale prospective studies to confirm its real-world utility.

Through the development of a nomogram, this study provides further empirical evidence for the clinical application of NETs in predicting deep vein thrombosis (DVT) in patients with hyperhomocysteinemia (hHcy). For instance: Patient A (Risk <0.1): MPO-DNA score = 10, CitH3 score = 14, cfDNA score = 12, Neutrophil score = 7, total points = 43, Risk <0.1. Patient B (Risk >0.9): MPO-DNA score = 45, CitH3 score = 20, cfDNA score = 55, Neutrophil score = 30, total points = 150, Risk >0.9.

Our study provides compelling evidence that NETs play a critical role in venous thrombosis development among hHcy patients, establishing NETs inhibition as a promising therapeutic strategy for DVT prevention and treatment. The therapeutic potential is supported by multiple lines of evidence: DNase I has been shown to effectively degrade the DNA backbone of NETs, leading to significant reductions in cf-DNA and MPO-DNA levels (Chen et al., 2023), while PAD4 inhibitors such as GSK484 and Cl-amidine can suppress NETs formation by blocking the critical histone citrullination process (Liu X. et al., 2021). Additionally, NADPH oxidase inhibitors and catalase demonstrate inhibitory effects on NETosis through ROS reduction (Fuchs et al., 2007), and various pathway-specific inhibitors including p38 MAPK inhibitors and HMGB1 antagonists have shown efficacy in modulating NETs-associated signaling pathways (Liu et al., 2024). These findings collectively highlight NETs as a multifunctional therapeutic target, offering multiple potential intervention points for antithrombotic therapy in hHcy-associated DVT. The diverse mechanisms of NETs inhibition - ranging from structural disruption to enzymatic blockade and signal pathway modulation - provide a robust foundation for developing novel treatment approaches that could complement existing antithrombotic strategies.

It is important to acknowledge several limitations in the current study. First, as a retrospective investigation, this research is inherently susceptible to selection bias and information bias. Second, while our findings are statistically significant, the sample size remains relatively limited due to strict inclusion criteria, necessitating future validation through larger-scale, multicenter prospective studies. Furthermore, although the model developed in this study has undergone internal validation, its robustness and generalizability still require further verification with external datasets. In our future research, we plan to conduct prospective large-scale studies to further validate the findings presented in this work. Finally, the prediction model was constructed using unimodal clinical parameters, whereas DVT pathogenesis involves complex multifactorial interactions. Future research directions should incorporate multimodal data (e.g., radiomics, genomics) to develop more comprehensive and precise predictive models.

## 5 Conclusion

hHcy-associated thrombogenesis is closely correlated with elevated NETs, suggesting that NETs may play a crucial role in the pathogenesis of hHcy-related DVT. In this study, we developed and validated a non-invasive clinical prediction model for thrombosis risk in hHcy patients. The detection of MPO-DNA complexes, CitH3, cfDNA, and neutrophil counts may serve as auxiliary diagnostic markers for DVT. Furthermore, pharmacological inhibition of NET formation represents a clinically feasible strategy for DVT prevention in hHcy patients, offering novel therapeutic perspectives.

## Data Availability

The datasets presented in this study can be found in online repositories. The names of the repository/repositories and accession number(s) can be found in the article/supplementary material.
